# Metachronous Krukenberg tumor from adenocarcinoma in a Meckel’s diverticulum: a case report

**DOI:** 10.1093/jscr/rjab374

**Published:** 2021-08-31

**Authors:** Ryotaro Sakio, Homare Ito, Gaku Ota, Makiko Tahara, Tomonori Yano, Koji Koinuma, Hisanaga Horie, Alan Kawarai Lefor, Hironori Yamamoto, Naohiro Sata

**Affiliations:** Department of Surgery, Division of Gastroenterological, General and Transplant Surgery, Jichi Medical University, Tochigi, Japan; Department of Surgery, Division of Gastroenterological, General and Transplant Surgery, Jichi Medical University, Tochigi, Japan; Department of Surgery, Division of Gastroenterological, General and Transplant Surgery, Jichi Medical University, Tochigi, Japan; Department of Surgery, Division of Gastroenterological, General and Transplant Surgery, Jichi Medical University, Tochigi, Japan; Department of Medicine, Division of Gastroenterology, Jichi Medical University, Tochigi, Japan; Department of Surgery, Division of Gastroenterological, General and Transplant Surgery, Jichi Medical University, Tochigi, Japan; Department of Surgery, Division of Gastroenterological, General and Transplant Surgery, Jichi Medical University, Tochigi, Japan; Department of Surgery, Division of Gastroenterological, General and Transplant Surgery, Jichi Medical University, Tochigi, Japan; Department of Medicine, Division of Gastroenterology, Jichi Medical University, Tochigi, Japan; Department of Surgery, Division of Gastroenterological, General and Transplant Surgery, Jichi Medical University, Tochigi, Japan

## Abstract

Adenocarcinoma in a Meckel’s diverticulum is rare and difficult to diagnose preoperatively. We report the first case of a metachronous Krukenberg tumor from adenocarcinoma in a Meckel’s diverticulum. A 45-year-old woman was admitted for recurrent abdominal pain. Computed tomography scan showed a lesion with contrast enhancement, and a Meckel’s diverticulum-associated tumor was suspected. Double-ballon enteroscopy revealed intestinal stenosis and biopsy showed adenocarcinoma. Operative findings showed a Meckel’s diverticulum with tumor. Histopathological evaluation revealed well-differentiated adenocarcinoma, interrupted by ectopic gastric mucosa, diagnosed as adenocarcinoma in a Meckel’s diverticulum. Two years postoperatively, a multi-cystic mass with contrast enhancement was observed in the pelvis on imaging evaluation and oophorectomy performed. Histological examination of the resected ovary showed proliferation of atypical glandular ducts, consistent with metastatic adenocarcinoma. This case demonstrates that adenocarcinoma in a Meckel’s diverticulum may result in distant metastases and requires appropriate follow-up.

## INTRODUCTION

Meckel’s diverticulum is the most common anomaly of the gastrointestinal tract which is a true diverticulum. The prevalence is estimated to be ~1.2–2.0% of the population [1, 2]. Only 2.0–4.0% of patients with a Meckel’s diverticulum present with symptoms and complications [3]. Meckel’s diverticulum-associated malignancy is very rare, reported to affect just 3.2% of symptomatic patients with Meckel’s diverticula [4]. Although Krukenberg tumor associated with adenocarcinoma in a Meckel’s diverticulum has been reported twice previously, these were synchronous lesions [5, 6]. To the best of our knowledge, this is the first report of a patient with a metachronous Krukenberg tumor from adenocarcinoma in a Meckel’s diverticulum.

## CASE REPORT

**Figure 1:**
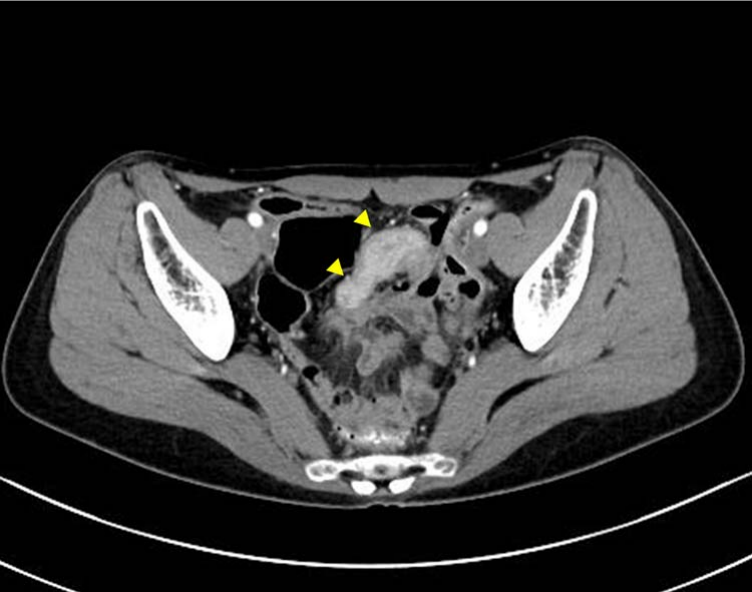
Computed tomographic scan showing a continuous lesion to the ileum with contrast enhancement (arrowheads).

**Figure 2:**
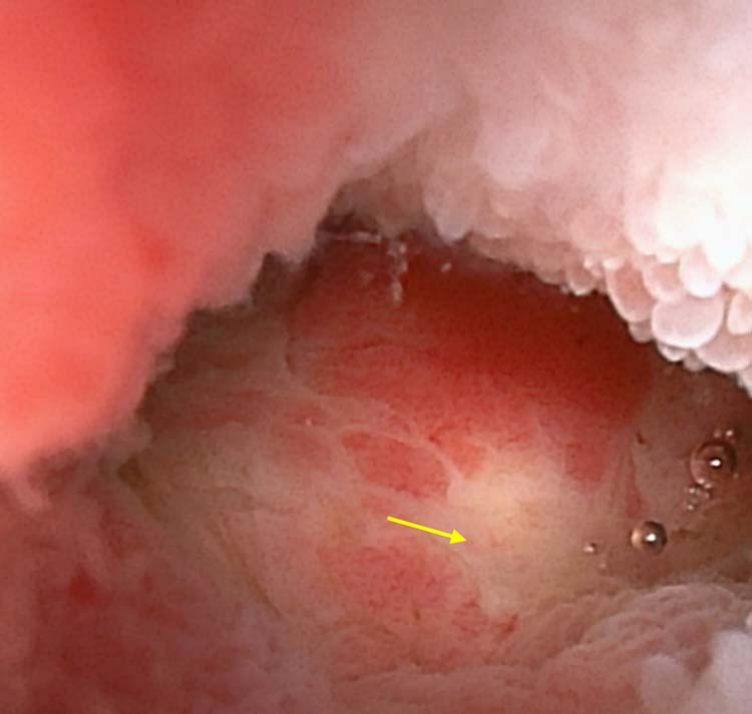
Double-ballon enteroscopy showing an ulcerated lesion (arrow) at 160 centimeters from the ileocecal valve.

**Figure 3:**
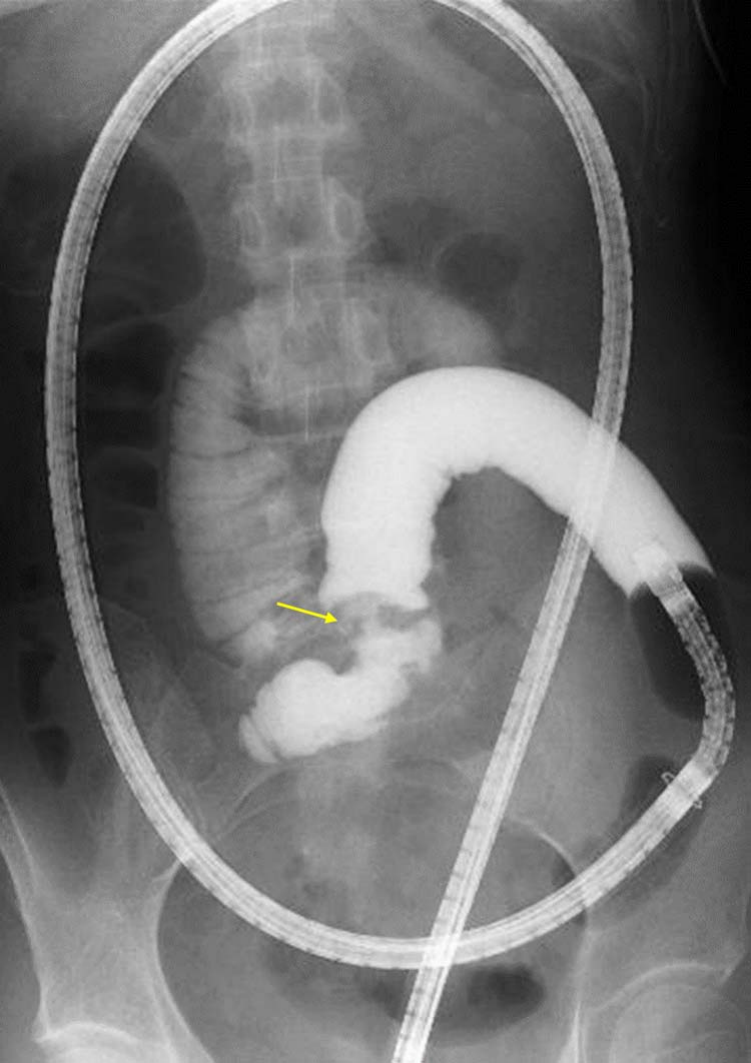
Double-ballon enteroscopy-assisted contrast study demonstrating intestinal stenosis involving a Meckel’s diverticulum (arrow).

**Figure 4:**
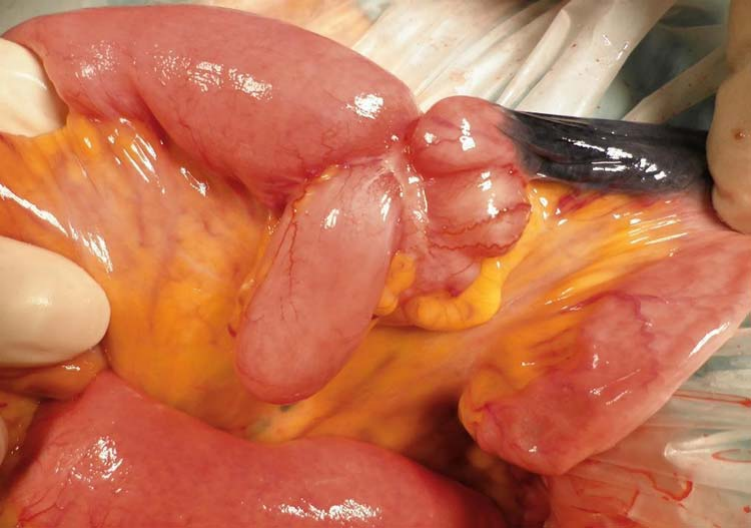
Operative findings showing tumor invasion involving a Meckel’s diverticulum at 160 centimeters from the ileocecal valve.

**Figure 5:**
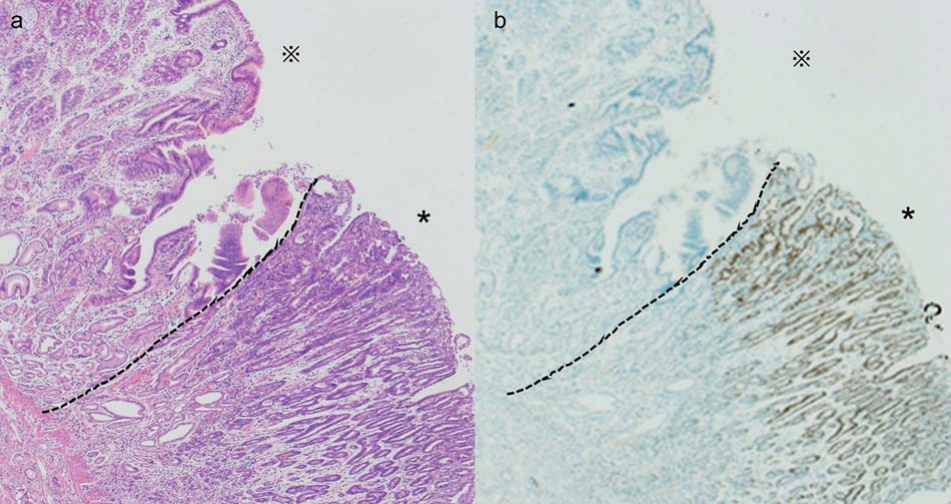
Histological evaluation of the resected specimen. (**a**) Hematoxylin and eosin stain showed a differentiated adenocarcinoma adjacent to the ectopic gastric mucosa (*: ectopic gastric mucosa, *: adenocarcinoma, ×40). (**b**) Immunohistochemical stain of CDX2 showed expression in a differentiated adenocarcinoma (*: ectopic gastric mucosa, *: adenocarcinoma, ×40)

A 45-year-old woman was admitted with recurrent abdominal pain. She had no significant past medical history. Physical examination showed mild tenderness in the lower abdomen. Laboratory findings were within normal limits, except for serum carbohydrate antigen 19-9 (58 U/mL). Computed tomography (CT) scan showed a continuous lesion to the ileum with contrast enhancement (Fig. 1). Retrograde double-ballon enteroscopy showed an ulcerated lesion at 160 centimeters from the ileocecal valve, and biopsy showed well-differentiated adenocarcinoma (Fig. 2). An enteroscopy-assisted contrast study revealed intestinal stenosis involving a Meckel’s diverticulum (Fig. 3). Based on these findings, we diagnosed adenocarcinoma in a Meckel’s diverticulum preoperatively. Intestinal resection with lymph node dissection was performed. Operative findings showed that the tumor invaded the Meckel’s diverticulum 160 centimeters from the ileocecal valve. No findings of abdominal dissemination were observed (Fig. 4). Histological examination of the resected specimen revealed a well-differentiated adenocarcinoma, interrupted by ectopic gastric mucosa (one side shown, Fig. 5a.).

Immunohistochemistry studies for caudal-type homeobox transcription factor 2 (CDX2), a marker for intestinal-type gastric cancer, showed strong expression in the adenocarcinoma (Fig. 5b). However, the diverticulum could not be clearly identified due to tumor invasion. The final pathologic stage was pT4aN0M0. These results strongly supported the possibility of intestinal-type adenocarcinoma with an ectopic gastric mucosal background, rather than primary small intestinal adenocarcinoma. She did well postoperatively and received adjuvant chemotherapy for 6 months.

**Figure 6:**
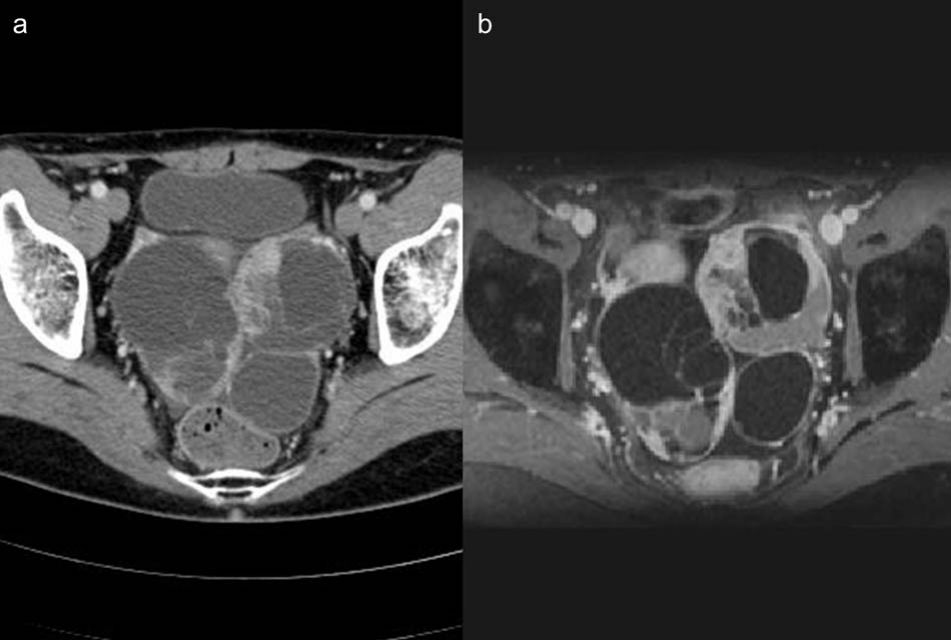
(**a**) Computed tomography scan showing a multi-cystic mass in the pelvis with a partial contrast effect. (**b**) Gadolinium-enhanced T2-weighted magnetic resonance image showing a solid mass with contrast effect in the cysts.

**Figure 7:**
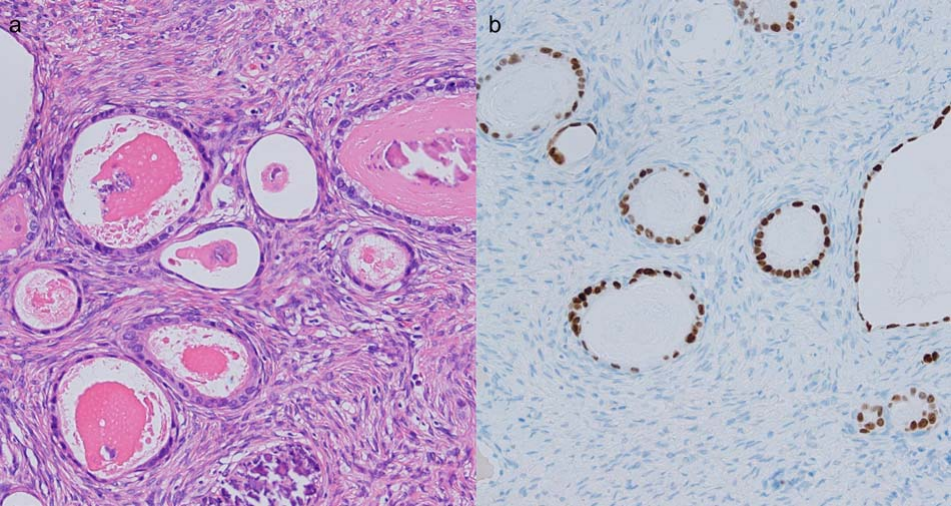
Histological evaluation of resected ovarian tissues. (**a**) Hematoxylin and eosin stain showed proliferation of atypical glandular ducts of various sizes, similar in morphology to those found in the intestinal tissue. (×200). (**b**) Immunohistochemical stain of CDX2 showed expression in glandular tissues (×200).

Two years after resection, the patient had no symptoms, but the serum carbohydrate antigen 19-9 became elevated (73 U/mL). CT scan showed a multi-cystic mass in the pelvis with some contrast enhancement (Fig. 6a). Gadolinium-enhanced T2-weighted magnetic resonance imaging revealed a solid mass with contrast enhancement in the cysts (Fig. 6b). Total hysterectomy with bilateral salpingo-oophorectomy was performed. Histological evaluation of the resected specimens showed a proliferation of atypical glandular ducts and expression of CDX2 in glandular tissues, similar to that found in the intestine (Fig. 7a and b). These findings established the diagnosis of a metachronous Krukenberg tumor from adenocarcinoma in a Meckel’s diverticulum. The patient remains free of disease after 1 year of follow-up.

## DISCUSSION

To the best of our knowledge, this is the first report of a metachronous Krukenberg tumor from adenocarcinoma in a Meckel’s diverticulum. The present patient confirms two important clinical points: (i) adenocarcinoma in a Meckel’s diverticulum may result in distant metastases and requires appropriate follow-up and (ii) double-balloon enteroscopic biopsy and contrast study are useful to establish a preoperative diagnosis.

Meckel’s diverticulum is the most frequent congenital anomaly of the gastrointestinal tract, which is a true diverticulum resulting from incomplete obliteration of the omphalomesenteric duct [1]. The prevalence of Meckel’s diverticula has been estimated to be ~1.2–2.0% [1, 2]. Approximately 2.0–4.0% of Meckel’s diverticula are reported to cause symptoms and complications [3]. Meckel’s diverticulum-associated malignancies are rare, accounting for ~3.2–5.1% of the symptomatic Meckel’s diverticula [4, 7]. The most frequently reported tumors associated with Meckel’s diverticula are neuroendocrine tumors, whereas others include gastrointestinal stromal tumors, adenocarcinoma and lymphoma [8]. Although precise data are not available, more adenocarcinomas have been reported in Japan than neuroendocrine tumors, so there may be regional and racial differences. Two patients with adenocarcinoma in a Meckel’s diverticulum with Krukenberg tumors have been reported, but both of these were synchronous lesions and there are no previous reports of metachronous metastases [5, 6]. One patient received adjuvant chemotherapy but relapsed with multiple peritoneal lesions 3 and 10 months later [5]. Thirunavukarasu *et al*. [8] reported that overall survival was dependent on the pathologic type and progression of disease (localized, regional and metastatic), suggesting that those with adenocarcinoma or distant metastases had the worst prognosis. In the present patient, intensive follow-up was necessary because of the presence of adenocarcinoma arising from ectopic gastric mucosa and metachronous distant metastases.

The preoperative diagnosis of tumors in Meckel’s diverticula is difficult. Previous reports demonstrate that tumors in Meckel’s diverticula were most often diagnosed after resection [7]. Double-ballon enteroscopy has been reported to be useful in patients undergoing evaluation for bleeding from Meckel’s diverticula [9]. Enteroscopy can allow diagnosis of the disease by direct vision, as well as facilitating biopsy of the lesion and, in some cases, treatment [9, 10]. In the present patient, we suspected a tumor arising in a Meckel’s diverticulum based on the CT scan findings and performed double-ballon enteroscopy. Enteroscopy showed an area of stenosis with ulceration, and involvement of the Meckel’s diverticulum in a contrast examination, allowing preoperative diagnosis of adenocarcinoma in a Meckel’s diverticulum.

In conclusion, we report a patient with a metachronous Krukenberg tumor from an adenocarcinoma in a Meckel’s diverticulum. Intensive follow-up is necessary because of the aggressive nature of the tumor in this patient. Double-ballon enteroscopy was useful to preoperatively establish the diagnosis.
